# Supplementary material for: Incidence of cervical, breast and colorectal cancers between 2010 and 2015 in people living with HIV in France
Source: PLoS One. 2022 Mar 25;17(3):e0261069. doi: 10.1371/journal.pone.0261069 (PMC8956191; doi:10.1371/journal.pone.0261069)
Supplement: Attachment — Submitted filename: Response to Reviewers.docx [file pone.0261069.s002.docx]

**REVIEW COMMENTS TO THE AUTHOR**

**REVIEWER #1:** Title: "Incidence of cervical, breast and colorectal cancer in people living with HIV between 2010 and 2015"

The manuscript presents a comprehensive study on a large study sample, regarding incidence trends for the period 2010-2015 for the three cancers included in the French organized screening program for cancer prevention: BC, CRC and ICC.

The study revealed an expected increase of ICC incidence in WLWH than in women in the general population, and also, present an interesting explanation for lower BC incidence rate.

Regarding ICC, it would be worth mentioned that Pap test is not having optimal sensitivity in detecting precancerous lesions of ICC, both in general population and in WLWH.

Organized national CC screening should be implemented worldwide, not only in France, with clinically validated HPV tests, able to detect HR HPV types.

It is known that in WLWH the risk for developing ICC developing is higher, as some studies detected that the risk for evolution to ICC was correlated with the multiple HR HPV types infections and with the number of sexual partners.

Global elimination of cervical cancer is a prioritized aim of WHO, and this could be achieved by two major strategies: stopping the HPV circulation by vaccination and offering HPV screening to those may have been infected before HPV circulation was stopped.

**Answer:** We thank the reviewer for these comments and suggestions. We added the following sentences in the Discussion section (lines 395-398):

*“****Currently, the screening methods for cervical cancer in France differ according to the age of the women. The HPV test, which looks for cervical infection by high-risk types of HPV, is now recommended instead of the Pap test every five years after a first negative result in women aged 30 to 65 years old (HAS guidelines, 2019)”.***

Moreover, we added the reference to the WHO guide for comprehensive cervical cancer control, which emphasizes the importance of early detection together with HPV vaccination (lines 398-402):

***“Global elimination of cervical cancer is a World Health Organization (WHO) priority, and screening for ICC must be part of a comprehensive preventive approach that includes HPV vaccination, the early treatment of pre-cancerous lesions, and the appropriate and rapid management of women diagnosed with ICC (WHO,*** ***Comprehensive cervical cancer control - A guide to essential practice, 2014)”.***

**REVIEWER #2:** Review PONE-D-21-14748 

The study has the good intention to provide statistic data for epidemiologic monitoring of cancers among PLWH due to deficient data on cancer incidence in France since 2009. While the study appears to be sound, the language is unclear, making it difficult to follow. The authors should revise the language to improve the flow and readability of the text. Secondly, the study did not clarify how to assign the cancer case that has double or triple cancer diagnosis during analysis.

**Answer:** As recommended by the reviewer, we modified several parts of the manuscript in order to improve clarity and comprehension. The entire manuscript has been thoroughly reviewed by a professional English mother-tongue copyeditor with over 15 years’ experience in the field.

With regard to double or triple cancer diagnosis, we better specified how these were treated in the analyses.

In the “Study population and study period” section, we specify that: “***For each of the three study analyses (one for each cancer type), the end of the study period was set at the first diagnosis of the specific cancer type for patients with at least one diagnosis of that cancer between 01 January 2010 and 31 December 2015, and at the last database update for all other patients, with censoring at 31 December 2015***”.

In the “Definition of cancer cases” section of the revised version of the manuscript, we added the following sentence: “***For each cancer type, only the first diagnosis during the study period was retained in the analysis. Prevalent cancer cases - defined here as diagnosis during the first 30 days after enrolment in Dat'AIDS - were excluded. Recurring and metastatic cancers were also excluded***”.

ICC, BC and CRC are the three most common targeted cancers by the nationally organized cancer-screening program in France. Furthermore, BC as the second common cancer in WLWH and the fifth death cause, the study rarely described and discussed BC data.

**Answer:** With regard to BC data reported in the Results section, we provide the overall incidence rate (lines 271-273):

***“Among the 13,543 women included, accounting for 56,150.6 person-years, the overall incidence rates between 2010-2015 of BC and ICC were 91.0 [95% CI: 69.1-119.7] and 28.5 [95% CI: 17.5-46.5] per 100,000 person-years, respectively (Table 2)”;***

and the results on the linear trend over the study period of the incidence rate per calendar year (lines 276-279):

***“The incidence rates per calendar year showed a non-significant linear trend over the study period for the three different cancers (p-value=0.071, 0.237, and 0.672, for BC, ICC, and CRC, respectively, Figs 1A-1C)”.***

In the revised version of the manuscript, the SIR results are presented more clearly (lines 308-310):

***“The incidence rate observed for BC was 44% lower (SIR=0.56, 95% CI: 0.42-0.73) overall in the study population than in the general population, and in all women sub-populations (Table 3)”.***

An entire paragraph in the Discussion section is dedicated to BC, including a possible explanation for the lower BC incidence rate we found in our cohort with respect to that of the general population (lines 354-376):

***“With regard to BC, the women in our study (both overall and in terms of each specific sub-population) had a lower incidence rate than women in the general population. The lower incidence rate in WLWH in our study is consistent with previous studies (32). One hypothesis for this is that ART targeting both viral and tumorigenic proteins may interfere with oncogenic pathways, reducing tumour progression (33). Another hypothesis is that cellular apoptosis is produced due to HIV binding to the receptor CXCR4 on breast epithelium (34). Among a total of 31,099 men in our study, only four BC cases were observed (these cases were not included in the incidence analyses). Due to very low incidence, BC screening awareness in men remains poor (35), which might lead to late diagnosis (36,37).***

***The higher incidence of BC observed in some sub-populations of WLWH may be explained, at least partially, by the HIV-related characteristics (nadir CD4 > 200 cells/mm3, HIV transmission through IVDU, HIV diagnosis before 2000) and absence of HCV coinfection.***

***In the general population, chronic HCV infection has not only been other associated with an increased risk of hepatocellular carcinoma, but also of other cancers (e.g., oesophagus, pancreas, prostate, thyroid, breast and oral cavity) (38,39). In our cohort, the ICC incidence rate and SIR, as well as the incidence rate of CRC were higher in HCV coinfected women than in women with no HCV coinfection. This was not observed for BC. This result could not be explored in detail due to the lack of HCV RNA data in our study. However, it may be associated with the fact that specific sub-populations, for example drug users, have vulnerabilities associated with an increased risk of HCV infection”.***

The last but the most important is that the same study using the same data source: the French Dat’AIDS cohort from 2010 to 2015 (Ref 17) have been published in 2020 with much more comprehensive analysis. Altogether, this study will not have a significant contribution to the field.

**Answer:** The previous study using the Dat’AIDS cohort was designed to describe the overall spectrum and incidence rates for AIDS-defining and non–AIDS-defining cancers, and for some specific most common cancers. However, it did not evaluate factors associated with the incidence of specific cancer types; incidences were only assessed for the entire study population and not for sub-populations (i.e., according to the different characteristics available in the database).

This is why the present study concentrated on the assessment of these factors, with a focus on the three cancers (BC, CRC and ICC) included in France’s national screening program for cancer prevention, with a view to encouraging these programs to target more specific at-risk populations.

Minor points to consider in subsequent versions:
1. significant numbers should be consistent as 0.00 or 0.0 throughout the manuscript.

**Answer:** With regard to the precision of the results, we used two numbers after the decimal point (0.00) for the standardized incidence ratios (with values ranging from 0 to 5.2), and a number after the decimal point (0.0) for the incidence rates per 100,000 person-years, as these had larger values (ranging from 0 to 128).

2. Be consistent with “CD4 cell count” and “HCV coninfection” not “HCV-coinfection” in the text.

**Answer:** Following the reviewers’ suggestion, we consistently used “CD4 cell count” and “HCV coinfection” throughout the manuscript.

3. Some statistic data in text are not match the data listed in Tables. For example, Page 4: Results: The incidence rate of CRC between 2010 and 2015 as 25.0 [95% confidence interval (CI): 18.6-33.4] (in text) vs 25.0 [18.7-33.5] (in Table 2).

**Answer:** Thank you for this remark. The correct value is 25.0 [18.7-33.5]. This was an error in the sentence in the Results section of the manuscript and has been corrected.

4. Tables are better to use the same format. For example, Table 2: 28.5 [17.5-46.5] vs Table 3: 1.93 [1.18 to 3.14].

**Answer:** We now use the same format for Tables 2 and 3.

5. Page 4: Results: Author represented the standardized incidence ratio of ICC and BC, instead of incidence rate in the manuscript.

**Answer:** The SIR are only given for ICC and BC, but the incidence rates are given for each of the three cancer types. This is why only the incidence rate for CRC is presented in the abstract.

We reworded the Methods section of the Abstract to improve clarity, as follows:

***“Standardized incidence ratios (SIR) for ICC and BC, and incidence rates for all three cancers were calculated overall and for specific sub-populations according to nadir CD4 cell count, HIV transmission category, HIV diagnosis period, and HCV coinfection.”.***

In the Methods section of the manuscript, we clarify the SIR definition and why the calculation of the SIR could not be performed for CRC, as follows (lines 208-223):

***“Specifically, we calculated the standardized incidence ratios (SIR) (see below) for ICC and BC, using indirect standardization (24), and their 95% confidence intervals based on a Poisson distribution. The SIR for CRC could not be calculated, because incidence rate data for the French general population were lacking (they were available only for colon, rectum, and anal cancers grouped together, but not for CRC without anal cancer). Data were aggregated by sex and age strata, using the following 5-year age intervals: [15-19], [20-24], [25-29], [30-34], [35-39], [40-44], [45-49], [50-54], [55-59], [60-64], [65-69], [70-74], [75-79], [80-84], [85-89], [90-94] and [≥ 95]. Incidence rates were computed for each sex-age stratum and compared with the related observed incidence rates in the general population.***

***The SIR represented the ratio between the incidence rate in our cohort and the incidence rate in the general population, after adjusting for sex and age. If the SIR was >1, then an excess risk of cancer existed in our cohort compared to the general population. More specifically, the incidence rate in the cohort was (SIR-1)*100 percent higher than in the general population. Conversely, if SIR was <1, then the incidence rate in the cohort was (1-SIR)*100 percent lower than in the general population”.***

6. Page 6: “the widespread use of ARV [9, 10],” should be “the widespread use of ART [9, 10],”

**Answer:** Thank you for this remark. We corrected this.

7. Page 13: The numbers in the text do not match with the numbers in Table 1: “and nadir CD4 were 601 [IQR: 413-810] and 223 [IQR: 97-350] cells/mm3, respectively.

Median CD4 count was 354 [IQR: 264-627] for patients with ICC, 717 [IQR: 408-913] for patients with BC and 446 [IQR: 271-659] for patients with CRC (Table 1).” VS Table 1 numbers: “and nadir CD4 were 223 [97-350] and 601 [413-810] cells/mm3, respectively. Median CD4 count was 354 [264-627] for patients with ICC, 718 [408-943] for patients with BC and 402 [271-630] for patients with CRC.

**Answer:** Again, our thanks for these corrections. The text now reads (lines 254-259):

*“****Median CD4 cell count and nadir CD4 cell count at the first diagnosis of each cancer type, or at the last available visit for patients with no cancer diagnosed during the study period, were 601 [IQR: 413-810] and 223 [IQR: 97-350] cells/mm3, respectively. Median CD4 cell count at the first diagnosis of each cancer type was 354 [IQR: 264-627] for patients with ICC, 718 [IQR: 408-943] for patients with BC and 402 [IQR: 271-630] for patients with CRC (Table 1)****”.*

8. Page 14: Table 2: IR for ICC & HCV coinfection is exactly same as IR for CRC (Women) & HCV coinfection: 59.1 [24.6-142.0]. Is it coincident or miss type the data for cancer case?

**Answer:** The two incidences are identical, because they correspond to the same number of cancer cases (n = 5) for ICC and CRC in women, observed for the same population followed over the same period (same number of person-years).

9. Page 15: Figure 1 has not been explained/presented well in the manuscript. The conclusion “The incidence rates per calendar year remained relatively stable over the study period for the three different cancers (Figs 1A-1C).” from Figure 1 is contradict with Figure 1 presented.

**Answer:** We now explain the method used and the results in Figure 1 in greater detail, as follows:

In the Methods section (lines 226-228):

***“We also computed the incidence rates per calendar year, and performed a χ2 test for linear trend to determine whether a significant trend occurred in new cancer cases over the study period. A p-value <0.05 was considered significant”.***

In the Results section (lines 276-279):

***“The incidence rates per calendar year showed a non-significant linear trend over the study period for the three different cancers (p-value=0.071, 0.237, and 0.672, for BC, ICC, and CRC, respectively, Figs 1A-1C)”.***

The first sentence of the Conclusion was modified as follows (lines 414-415):

***“In conclusion, the present study did not show any significant linear trend between 2010 and 2015 in the incidence rates of the three cancer types explored in PLWH”.***

10. Page 15: The section: “SIR for ICC and BC, overall and according to patient profile”: it is supposed to present SIR data from Table 3. However, “incident rate” was in the text instead. Those are two different analysis definition.

**Answer:** In this section we presented SIR data from Table 3, which allowed us to compare incidence rates in the study population and in the general population. To clarify the relationship between SIR and incidence rates, we added the following sentences in the Methods section (lines 218-223):

***“The SIR represented the ratio between the incidence rate in our cohort and the incidence rate in the general population, after adjusting for sex and age. If the SIR was >1, then an excess risk of cancer existed in our cohort compared to the general population. More specifically, the incidence rate in the cohort was (SIR-1)*100 percent higher than in the general population. Conversely, if SIR was <1, then the incidence rate in the cohort was (1-SIR)*100 percent lower than in the general population”.***

11. Page 15: “The ICC incidence rate in the women in our study population was 93% higher (SIR=1.93; 95% CI, 1.18-3.14) than that in women in the general population (Table 3). However, it was 44% lower (SIR, 0.56, 95% CI: 0.42-0.73) for BC.” The calculation/explanation is needed for the results of 93% higher and 44% lower.

**Answer:** In the Methods section, we now explain the definition of the SIR and the comparison of incidence rates in the study population and in the general population, as follows (lines 218-223):

***“The SIR represented the ratio between the incidence rate in our cohort and the incidence rate in the general population, after adjusting for sex and age. If the SIR was >1, then an excess risk of cancer existed in our cohort compared to the general population. More specifically, the incidence rate in the cohort was (SIR-1)*100 percent higher than in the general population. Conversely, if SIR was <1, then the incidence rate in the cohort was (1-SIR)*100 percent lower than in the general population”.***

12. Page 16: The study claimed that “This is the first study on incidence trends for the period 2010-2015 for the three cancers included in the French organized screening program for cancer prevention.” The similar study has already published in 2020 as in reference 17.

**Answer:** We corrected the first sentence of the Discussion section as follows (lines 319-321):

***“This study evaluated the incidence rates for the period 2010-2015 for ICC, BC and CRC, which are the three cancers included in the French national screening program for cancer prevention”.***
